# Vascular endothelial growth factor A inhibition remodels the transcriptional signature of lipid metabolism in psoriasis non‐lesional skin in 12 h ex vivo culture

**DOI:** 10.1002/ski2.471

**Published:** 2024-10-26

**Authors:** Andrea Luengas‐Martinez, Dina Ismail, Ralf Paus, Helen S. Young

**Affiliations:** ^1^ Centre for Dermatology Research and Manchester Academic Health Science Centre The University of Manchester Manchester UK; ^2^ Dr. Philip Frost Department of Dermatology and Cutaneous Surgery University of Miami Miller School of Medicine Miami Florida USA; ^3^ Monasterium Laboratory Muenster Germany

## Abstract

**Background:**

Vascular endothelial growth factor A (VEGF‐A)‐mediated angiogenesis is involved in the pathogenesis of psoriasis. VEGF‐A inhibitors are widely used to treat oncological and ophthalmological diseases but have not been used in psoriasis management. The molecular mechanisms underlying the effects of VEGF‐A inhibition in psoriatic skin remain unknown.

**Objectives:**

To identify the genes and canonical pathways affected by VEGF‐A inhibition in non‐lesional and plaque skin ex vivo.

**Methods:**

Total RNA sequencing was performed on skin biopsies from patients with psoriasis (*n* = 6; plaque and non‐lesional skin) and healthy controls (*n* = 6) incubated with anti‐VEGF‐A monoclonal antibody (bevacizumab, Avastin®) or human IgG_1_ isotype control for 12 h in serum‐free organ culture. Differentially expressed genes between paired control and treated samples with adjusted *p*‐values <0.1 were considered significant. Gene ontology and ingenuity pathway analysis was used to identify enriched biological processes, canonical pathways and upstream regulators.

**Results:**

VEGF‐A inhibition upregulated the expression of genes involved in lipid metabolism. Pathway enrichment analysis identified the activation of pathways involved in fatty acids and lipid biosynthesis and degradation in non‐lesional skin and ferroptosis in plaque skin. VEGF‐A inhibition downregulated endothelial cell apoptosis in non‐lesional psoriasis skin and members of the interferon family were identified as potential regulators of the effects of VEGF‐A inhibition in non‐lesional skin.

**Conclusion:**

Early response to VEGF‐A inhibition is associated with changes in lipid metabolism in non‐lesional psoriasis skin and cellular stress in psoriasis plaque. More investigation is needed to validate these findings.

## Graphical abstract

Vascular endothelial growth factor A (VEGF‐A)‐mediated angiogenesis is central to the pathogenesis of psoriasis. Although, the molecular mechanisms underlying the effects of VEGF‐A inhibition in psoriatic skin remain unknown. Here we determine that VEGF‐A inhibition remodels the transcriptional signature of lipid metabolism in psoriasis non‐lesional skin in ex vivo culture. More investigation is required as this may provide an opportunity to enhance cardiovascular risk management in psoriasis.

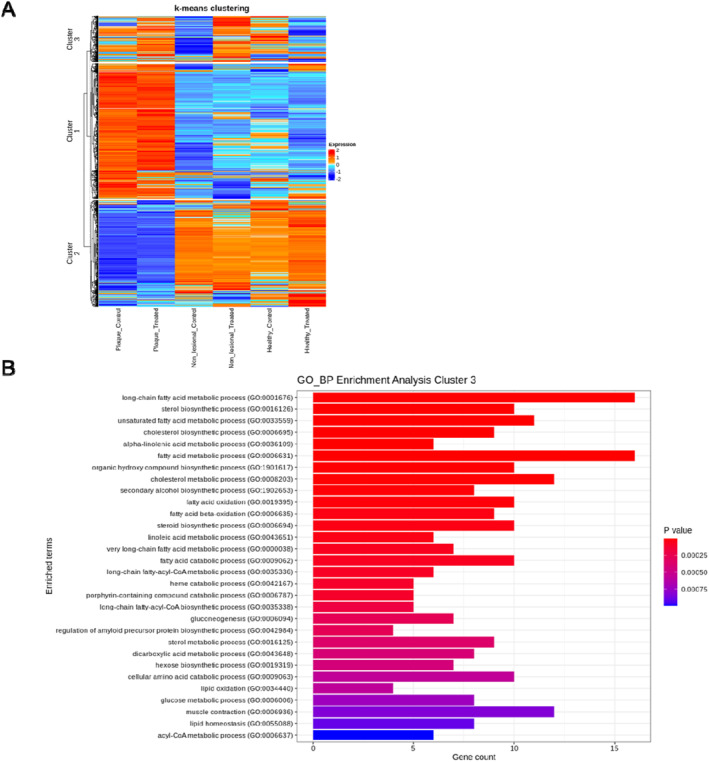

**What is already known?**

- Vascular endothelial growth factor A (VEGF‐A)‐mediated angiogenesis participates in the pathogenesis of psoriasis.

**What does this study add?**

- VEGF‐A inhibition alters lipid metabolic pathways in psoriasis skin ex vivo.

**What is the translational message?**

- Targeting VEGF‐A may provide an opportunity for cardiovascular risk management in psoriasis.

## 1 INTRODUCTION

Psoriasis, a chronic immune‐mediated systemic disease that affects 2%–4% of the world's population, is associated with comorbidities including cardiovascular disease and metabolic syndrome as well as reduced quality of life.^1^, ^2^, ^3^ Indeed, the prevalence of risk factors for cardiovascular diseases including diabetes, hypertension, obesity and hyperlipidaemia is higher in patients with psoriasis than in healthy people.^3^

The pathogenesis of psoriasis is characterized by an enhanced immune response, epidermal keratinocyte hyperproliferation and aberrant angiogenesis.^4^, ^5^ Inflammatory‐type angiogenesis is observed in patients with psoriasis and consists of blood vessel dilation, elongation and increased permeability in psoriasis plaques rather than new blood vessel formation.^6^ Angiogenesis is predominately mediated by vascular endothelial growth factor A (VEGF‐A), which is overexpressed in the skin and plasma of patients with psoriasis. Bevacizumab, a humanized monoclonal antibody, binds to VEGF‐A preventing the activation of VEGF‐A signalling pathways.^7^ Bevacizumab was the first anti‐VEGF‐A biological therapy approved for clinical application and is the most studied VEGF‐A inhibitor.^8^, ^9^ Since its approval by the Food and Drug Administration in 2004,^9^ bevacizumab has been widely used to treat different types of cancer and age‐related macular degeneration.^10^, ^11^, ^12^, ^13^, ^14^ Preclinical^15^, ^16^, ^17^, ^18^, ^19^, ^20^, ^21^ and anecdotal evidence from case reports of resolution of psoriasis whilst on anti‐VEGF‐A treatments,^22^, ^23^, ^24^, ^25^, ^26^, ^27^, ^28^ support the potential of VEGF‐A‐targeting therapies for psoriasis.^29^ Previously we showed that VEGF‐A inhibition with bevacizumab (Avastin®; Roche) downregulated angiogenesis in psoriasis plaque skin ex vivo.^30^ However, the effects of VEGF‐A inhibition on the transcriptional landscape of psoriasis skin remain underexplored.

Here, using RNA sequencing we interrogate the transcriptome in plaques of psoriasis, non‐lesional and healthy skin incubated with bevacizumab for 12 h ex vivo, postulating that VEGF‐A inhibition influences key transcriptional pathways involved in psoriasis pathogenesis. Using an established preclinical model of psoriasis, we demonstrate that VEGF‐A inhibition alters the transcription of genes involved in lipid metabolism and that the interferon (IFN) family mediate these effects in non‐lesional skin from patients with psoriasis.

## 2 MATERIALS AND METHODS

### 2.1 Human volunteers

Twelve individuals (6 patients with chronic plaque psoriasis and 6 subjects without psoriasis) were recruited from the dermatology department of Salford Royal Hospital, Manchester, UK. Potential donors were excluded from the study if they had used topical treatment within 4 weeks or systemic treatments within 12 weeks of enrolment, had inflammatory arthropathy or were taking COX‐1 or COX‐2 non‐steroidal anti‐inflammatory drugs which can inhibit angiogenesis by blocking VEGF‐A‐induced signal transduction.^31^, ^32^ The study was approved by the UK Health Research Authority (15/NW/0585, 09/H/1011/43) and adhered to the Declaration of Helsinki Guidelines. All subjects gave written informed consent.

### 2.2 Skin sampling

Skin punch biopsies (3 mm) were collected from all donors. Those with psoriasis had biopsies taken from plaques of psoriasis and from non‐lesional skin (at least 5 cm away from plaques). Anatomical site‐to‐site variations were minimized by taking the biopsies from the same body site.

### 2.3 Human skin organ culture

Skin biopsies were incubated in supplemented EpiLife media (MEPI500CA, Gibco™; Thermo Fisher Scientific) with a T cell activation mix for 12 or 72 h. Experimental conditions for organ culture were iterated from our previously published optimizing experiment using biopsies from healthy skin.^33^ Skin biopsies were incubated either with 0.8 mg/mL of bevacizumab or 0.8 mg/mL of IgG_1_ isotype control (BioXCell, 2BScientific Ltd.) at 37°C and 5% CO_2_. After 12 h, biopsies were snap frozen in liquid nitrogen and stored at −80°C until RNA processing.

### 2.4 RNA extraction

Skin biopsies were soaked in prechilled RNA*later*®‐ICE (AM7030, Thermo Fisher Scientific) for 16 h at −20°C before RNA extraction. RNA was extracted using the RNeasy® Plus Universal Mini Kit (73404, QIAGEN®) and RNA samples were stored at –80°C until sequencing (Figure S1).

### 2.5 Library preparation for RNA sequencing

Sequencing library preparation was performed by The University of Manchester Genomic Technologies Core Facility. Complementary DNA libraries were generated using the Illumina TruSeq® Stranded mRNA platform and RNA sequencing was performed on an Illumina HiSeq4000 instrument (Illumina). Paired‐end sequencing (76 + 76 cycles) resulted in ∼33 million paired‐end reads per sample. The quality of RNA‐Seq reads was assessed using FastQC (v0.11.3), FastQ Screen (v0.14.0) and FastqStrand (v0.0.7). Raw data were converted to FASTQ files using Illumina's bcl2fastq software (v2.20.0.422).

### 2.6 Data analysis and statistics

Raw sequencing data were trimmed with a Trimmomatic filter (v0.39). Sequencing reads were mapped against the human genome (hg38/39gtf) in STAR. Reads were counted and normalized in R using the biomaRt (v2.48.3) package. Principal component analysis was performed in DESeq2 (v1.32.0 R4.1.1).

### 2.7 Selection of differentially expressed genes

Paired gene expression was compared between plaque‐treated and control, non‐lesional‐treated and control and healthy‐treated and control samples. Log2 fold change (FC) was calculated for each gene and *p‐*values were corrected for multiple testing using the Benjamini–Hochberg approach in DESeq2, which controls the false discovery rate. Genes with adjusted *p*‐values <0.1 and absolute log2FC > 1 were considered significant.

### 2.8 GO enrichment analysis

Differentially expressed genes (DEGs; *p‐*value <0.05) between treated and control samples across the groups were used as input for clustering to define the prevalent gene expression patterns. The Silhouette method was used to evaluate cluster quality using the k‐means algorithm. Gene ontology (GO) enrichment analysis was performed to identify overrepresented biological processes in our dataset.

### 2.9 Ingenuity pathway analysis

The list of DEGs (containing the gene identifiers and their corresponding FC values) was uploaded into QIAGEN's ingenuity pathways analysis (IPA v22.0, QIAGEN Redwood City, www.qiagen.com/ingenuity) and a filter was applied for *p*‐value <0.05. Enrichment of ‘canonical pathways’ with a *Z*‐score >2 or < −2 were considered for analysis. Upstream regulator analysis was conducted using a *p*‐value of overlap<0.01 and *Z*‐score >2 for analysis.

### 2.10 Immunofluorescence staining and quantification

Skin biopsies incubated for 72 h were used for immunofluorescence staining. Biopsies were embedded in optimal cutting temperature gel, snap frozen in liquid nitrogen and stored at −80°C until cryosectioning. Cryosections (8‐μm) were affixed to Superfrost Plus slides (Menzel‐Glaser, ThermoFisher Scientific). Double immunofluorescence staining mouse anti‐CD31 (clone JC/70A, M0823, DAKO, 1:100)/rabbit anti‐cleaved caspase (clone Asp175, 9661, Cell signalling, 1:100) was used. DAPI was used to stain nuclei. Secondary antibodies used were goat anti‐rabbit 488 Alexa Fluor (AF; 1:200), and goat anti‐mouse 594 AF (1:200). Slides were mounted using Fluoromount mounting medium (S3023, Dako) and were photographed using a 3D Histec Pannoramic250 Slide scanner (Leica Biosystems). Quantitative analysis was performed on QuPath v0.1.2.

## 3 RESULTS

### 3.1 Demographics of patients with psoriasis and healthy volunteers

Skin samples were taken from 6 patients with psoriasis (5 women and 1 man), having a median age of 46.5 years (interquartile range [IQR] 27.75), median Psoriasis Area Severity Index of 12.65 (IQR 9.8), the median age of psoriasis onset 24 years (IQR 17) and 50%; *n* = 3 had a family history of psoriasis. Skin samples were taken from 6 healthy matched volunteers with a median age of 42.5 years (IQR 14.5; Table 1).

**TABLE 1:** Demographic details and clinical characteristics of volunteers.

Patients with psoriasis							
Volunteer ID	PS1	PS2	PS3	PS4	PS5	PS6	Median (IQR)
Sex	F	F	F	F	F	M	
Ethnicity	White	White	White	White	White	White	
Age (years)	42	51	57	61	28	31	46.5 (27.75)
Age of onset	11	24	24	58	19	26	24 (17)
Disease duration	31	28	33	3	9	5	18.5 (27)
Family history	Yes	Yes	No	No	No	Yes	
Severity	10/10	8/10	3/10	3/10	5/10	5/10	5 (5.5)
PASI	16	21.8	6.6	8.5	9.3	16.5	12.65 (9.8)
PEST	4/5	2/5	0/5	0/5	3/5	2/5	2 (3.25)
DLQI	25/30	5/30	10/30	14/30	13/30	25/30	13.5 (16.25)
Finger nails	Yes (3)	No	No	No	Yes (3)	Yes (10)	1.5 (4.75)
Toe nails	Yes (3)	Yes (10)	No	No	Yes (2)	Yes (1)	1.5 (4.75)
Height (m)	1.72	1.67	1.69	1.55	1.72	1.88	1.7 (0.12)
Weight (kg)	101.4	119	85.4	78.4	111.1	136.2	106.3 (39.65)
BMI	34.2	42.6	29.9	32.6	37.5	38.5	35.91 (7.62)
SBP (mm Hg)	116	129	177	131	136	128	130.5 (21.3)
DBP (mm Hg)	77	84	107	82	80	84	83 (10.5)

Healthy volunteers							
Volunteer ID	H1	H2	H3	H4	H5	H6	Median (IQR)
Sex	F	F	M	M	F	F	
Ethnicity	White	White	White	White	White	White	
Age	38	49	46	39	54	29	42.5 (14.5)
Height (m)	1.6	1.6	1.82	1.8	1.6	1.6	1.68 (0.18)
Weight (kg)	102.4	92	121.8	96.3	66.6	65	96.3 (32.8)
BMI	39	33.8	36.7	29.4	23.6	23.3	33.79 (11.39)
SBP (mm Hg)	110	138	126	124	98	119	124 (22)
DBP (mm Hg)	80	78	100	86	68	77	80 (14.75)

*Note*: Values are expressed as median (range) unless otherwise noted.

Abbreviations: BMI, body mass index; DBP, diastolic blood pressure; DLQI, Dermatology Life Quality Index; H, healthy; IQR, interquartile range (25th–75th percentiles); PASI, Psoriasis Area Severity Index; PEST, Psoriasis Epidemiology Screening Tool; PS, Psoriasis; SBP, systolic blood pressure.

### 3.2 VEGF‐A inhibition blocked free VEGF‐A in organ culture

First, the ability of bevacizumab to block VEGF‐A in organ‐cultured skin was confirmed. VEGF‐A was undetectable at 12 h in the culture supernatant of plaque, psoriasis non‐lesional and healthy skin incubated with 0.8 mg/mL of bevacizumab (Figure S2). At 12 h, VEGF‐A levels in culture media of isotype control‐treated plaque were significantly higher than those of isotype control‐treated non‐lesional (**p < 0.01) and healthy skin (**p < 0.01, Figure S2), establishing that bevacizumab blocked free VEGF‐A in skin organ culture.

### 3.3 Psoriasis plaque, non‐lesional and healthy skin maintained their phenotype in organ culture

Principal components analysis of global transcriptional profiles showed that variation between the samples was primarily based on disease status (Figure S3). Non‐lesional skin clustered with healthy skin irrespective of treatment status (Figure S3), highlighting the differences between plaque and non‐lesional skin.^34^, ^35^ This demonstrated that organ‐cultured plaque, non‐lesional and healthy skin explants maintained their biological phenotype during 12 h ex vivo organ culture.

### 3.4 VEGF‐A inhibition induced differential gene expression in psoriasis plaque, psoriasis non‐lesional and healthy skin

The effects of VEGF‐A inhibition on the transcriptome of plaque, non‐lesional and healthy skin were assessed by comparing changes in transcript abundance between control and treated paired samples. Overall, we identified 43 DEGs (36 upregulated and 7 downregulated) in plaque, 276 DEGs (196 upregulated and 80 downregulated) in non‐lesional and 532 DEGs (272 upregulated and 260 downregulated) in healthy skin in response to VEGF‐A inhibition (*p*‐adjusted value <0.1; Figure S4). There was an overlap of 7 DEGs between plaque and non‐lesional skin, which were involved in chain elongation of fatty acids (*PECR*, *ELOVL3*),^36^ IL‐17A immune responses (*DEFB4A*)^37^ and inflammatory responses (*SPINK7* ^38^, ^39^; Table S1). This demonstrated that VEGF‐A inhibition induced differential effects in the transcriptome of plaque and non‐lesional skin ex vivo.

Genes with an absolute log2FC below 1 were filtered out leaving a total of 6 DEGs (5 upregulated and 1 downregulated) in plaque (Table S2), 75 DEGs (72 upregulated and 3 downregulated) in non‐lesional (Table S3) and 42 DEGs (3 upregulated and 39 downregulated) in healthy skin (Table S4) in response to VEGF‐A inhibition (Table 2 contains a summary of the top DEGs in plaque and non‐lesional skin). This implied that VEGF‐A inhibition had a greater impact on the transcriptome of non‐lesional skin compared to healthy and plaque skin ex vivo.

**TABLE 2:** Summary of top 15 DEGs in non‐lesional and plaque skin.

Gene	Log2FC	Adjusted *p*‐value
Psoriasis plaque skin		
*LINC02404*	−1.9	0.01
*SLCO4C1*	1.57	0.02
*ELOVL3*	1.42	0.06
*COCH*	1.31	0.01
*FADS2*	1.29	0.02
*FBP1*	1.29	0.02
Psoriasis non‐lesional skin		
*PADI4*	3.17	0.02
*SEC14L4*	3.08	0.00001
*THRSP*	2.95	2.08E‐07
*MOGAT2*	2.84	3.96E‐08
*IL12A‐AS1*	2.79	0.04
*MOGAT1*	2.78	0.002
*ACSM6*	2.63	0.00009
*CUX2*	2.58	2.01E‐07
*PCK1*	2.58	0.05
*METTL7B*	2.47	0.05
*LINC01612*	2.43	0.01
*SLC25A18*	2.34	0.008
*AGR2*	2.3	0.0003
*RNASE13*	2.23	0.06
*ELOVL3*	2.22	0.07

*Note*: DEGs with *p*‐value <0.1 and log2FC > 1 were considered significant. The DEGs were sorted by absolute Log2FC.

Abbreviation: DEGs, differentially expressed genes.

### 3.5 VEGF‐A inhibition altered lipid metabolism gene expression in psoriasis non‐lesional skin

**FIGURE 1:**
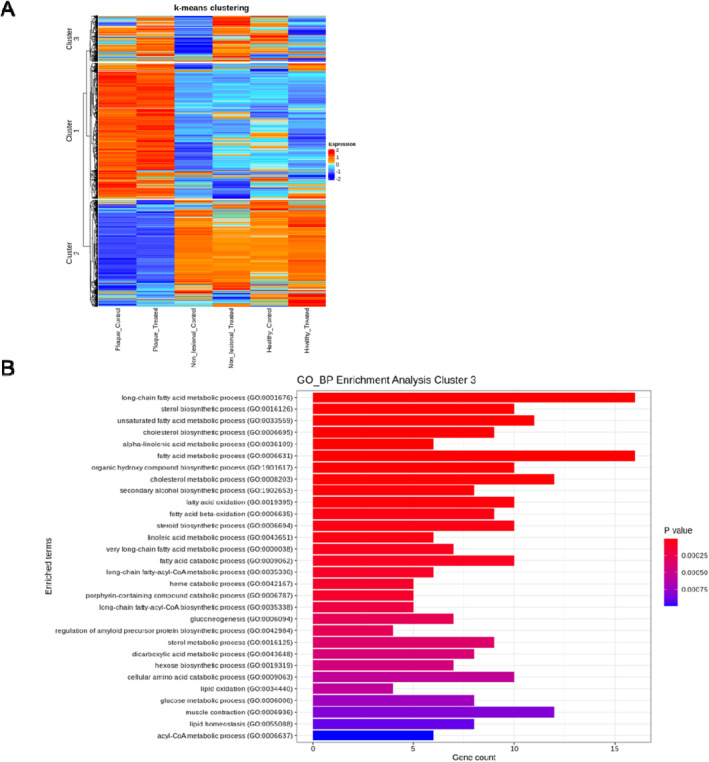
VEGF‐A inhibition upregulates the expression of genes involved in lipid metabolic processes in non‐lesional psoriasis skin. (a) K‐means clustering was used to generate a heat map containing the differentially expressed genes in healthy, non‐lesional and plaque skin in response to VEGF‐A inhibition. The genes in cluster 1 and cluster 2 did not show a response to VEGF‐A inhibition. Cluster 1 corresponds to the genes that were upregulated in plaque and downregulated in non‐lesional and healthy skin and cluster 2 corresponds to the genes that were upregulated in non‐lesional and healthy and downregulated in plaque skin, irrespective of anti‐VEGF‐A treatment. In contrast, VEGF‐A inhibition upregulated the expression of genes in cluster 3 in non‐lesional psoriasis skin. This response was not matched by plaque or healthy skin. (b) Gene ontology enrichment analysis shows that biological processes associated with lipid metabolism were significantly represented by genes in cluster 3. Red indicates upregulation, and blue indicates downregulation. VEGF‐A, vascular endothelial growth factor A.

To further analyse the effects of VEGF‐A inhibition on gene expression, a k‐means clustering was performed on the 3981 DEGs that passed the cut‐off (*p*‐value <0.05) for plaque, non‐lesional and healthy skin. K‐means clustering divided the DEGs into 3 clusters (cluster 1 = 1873 genes; cluster 2 = 1474 genes; cluster 3 = 634 genes). VEGF‐A inhibition did not alter gene expression in clusters 1 and 2 but upregulated the expression of genes in cluster 3 in non‐lesional and to a lower extent in plaque skin (Figure 1a).

GO functional analysis identified an enrichment of genes in cluster 3 in biological processes involved in lipid metabolism including long‐chain fatty acid, fatty acid and cholesterol metabolic processes (Figure 1b). Many of the genes affected by VEGF‐A inhibition in non‐lesional skin were associated with lipid metabolism (Table 3), suggesting that VEGF‐A inhibition predominately affects lipid metabolism in psoriasis non‐lesional skin.

**TABLE 3:** Differentially expressed genes involved in lipid metabolism in non‐lesional psoriasis skin.

Gene	Log2FC	Full name	Role
SEC14L4	3.08	SEC14 like lipid binding 4	Participates in the transport of hydrophobic ligands
THRSP	2.95	Thyroid hormone responsive	Controls tumour lipid metabolism
MOGAT2	2.84	Monoacylglycerol O‐acyltransferase 2	Catalyses the synthesis of diacylglycerol
MOGAT1	2.78	Monoacylglycerol O‐acyltransferase 1	Catalyses the synthesis of diacylglycerol
ACSM6	2.63	Acyl‐CoA synthetase medium chain family member 6	Involved in acyl‐CoA metabolism and fatty acid biosynthesis
METTL7B	2.47	Methyltransferase like 7B	Involved in methylation
ELOVL3	2.22	ELOVL fatty acid elongase 3	Catalyses the first reaction of the long‐chain fatty acids elongation cycle
CYP4F8	2.17	Cytochrome P450 family 4 subfamily F member 8	Catalyses reactions involved in drug metabolism and synthesis of cholesterol, steroids and other lipids
DHRS2	2.03	Dehydrogenase/reductase 2	Metabolises steroid hormones, prostaglandins, retinoids, lipids and xenobiotics
APOC1	1.88	Apolipoprotein C1	Role in HDL and VLDL metabolism and inhibition of cholesterol ester transfer protein in plasma
ACOX2	1.6	Acyl‐CoA oxidase 2	Involved in the degradation of long branched fatty acids and bile acid intermediates
HAO2	1.57	Hydroxyacid oxidase 2	Catalyses the oxidation of medium and long‐chain hydroxyacids. Contributes to the pathway of fatty acid α‐oxidation
ALOX15B	1.55	Arachidonate 15‐lipoxygenase type B	Involved in the production of fatty acid hydroperoxides
FAR2	1.35	Fatty acyl‐CoA reductase 2	Involved in wax biosynthesis wherein fatty acids are converted to fatty alcohols
PECR	1.32	Peroxysomal trans‐2‐enoyl‐CoA reductase	Participates in chain elongation of fatty acids
PLA2G7	1.21	Phospholipase A2 group VII	Involved in phospholipid catabolism during inflammatory and oxidative stress response
PLIN5	1.21	Lipid storage droplet protein 5	Coat intracellular lipid storage droplets and protect them from lipolytic degradation
GPD1	1.04	Glycerol‐3‐phosphate dehydrogenase 1	Link between carbohydrate metabolism and lipid metabolism
FAXDC2	1.03	Fatty acid hydroxylase domain containing 2	Involved in positive regulation of protein phosphorylation

Abbreviations: HDL, high density lipoprotein; VLDL, very low density lipoprotein.

Differentially expressed gene sets (*p‐value <0.05) from plaque, non‐lesional and healthy skin incubated with bevacizumab were compared to isotype control‐treated samples in IPA software to determine which canonical pathways were affected by VEGF‐A inhibition. A total of 1 inhibited and 39 activated pathways were identified in non‐lesional skin (Table S5). Most of these pathways were involved in fatty acid/lipid degradation (fatty acid β‐oxidation I, fatty acid α‐oxidation, mitochondrial L‐carnitine shuttle pathway and phospholipases) and fatty acid/lipid biosynthesis (superpathway of cholesterol biosynthesis, γ‐linoleate biosynthesis II, cholesterol biosynthesis I, cholesterol biosynthesis II, cholesterol biosynthesis III, stearate biosynthesis I, fatty acid activation and oleate biosynthesis II).

**FIGURE 2:**
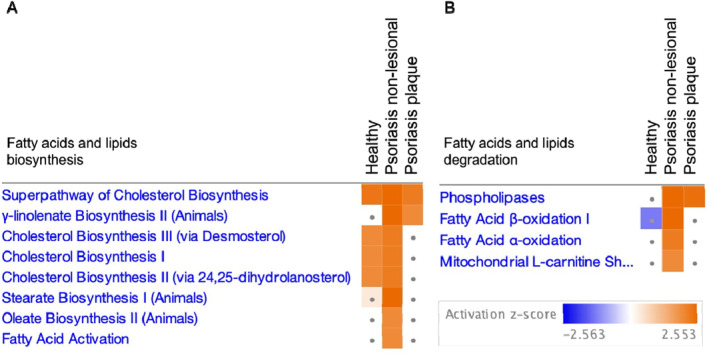
Comparison of pathways involved in lipid metabolism between healthy, psoriasis non‐lesional and psoriasis plaque skin. Canonical pathways in the categories of fatty acids and lipid biosynthesis and fatty acids and lipid degradation were compared using the comparison tool of ingenuity pathway analysis software. (a) Canonical pathways in the category of fatty acids and lipids biosynthesis that were enriched following bevacizumab treatment in healthy, psoriasis non‐lesional and psoriasis plaque skin. (b) Pathways in the category of fatty acids and lipids degradation that were enriched following bevacizumab treatment in healthy, psoriasis non‐lesional and psoriasis plaque skin. Pathways were sorted using the following criteria: *p*‐value <0.05, absolute *Z*‐score >2. The grey dot represents pathways that did not pass the *Z*‐score cut‐off. Orange means predicted activation and blue means predicted inhibition.

A comparison of the effects of VEGF‐A inhibition on pathways involved in fatty acid/lipid biosynthesis and degradation in plaque, non‐lesional and healthy skin (Figure 2), revealed that VEGF‐A inhibition altered these pathways in both non‐lesional and plaque skin but had its greatest effect in non‐lesional skin.

### 3.6 The interferon family mediated the effects of VEGF‐A inhibition in non‐lesional skin

Next, we used upstream regulatory analysis^40^ to identify upstream regulators responsible for gene expression changes in non‐lesional skin. We identified 239 upstream regulators in non‐lesional skin (Table S6; *p*‐value of overlap<0.01; absolute *Z*‐score >2). The top upstream regulators included members of the IFN family such as the cytokines IFN Lambda 1 (IFNL1), IFN Gamma (IFNG) and IFN Alpha 2 (IFNA2); transcription regulators such as interferon regulatory factor 7 (IRF7), IRF1 and signal transducer and activator of transcription 1 (STAT1) and the enzyme immunity related GTPase M. This suggested that the IFN family mediates changes in non‐lesional skin in response to VEGF‐A inhibition.

### 3.7 VEGF‐A inhibition decreased endothelial cell apoptosis in non‐lesional skin

**FIGURE 3:**
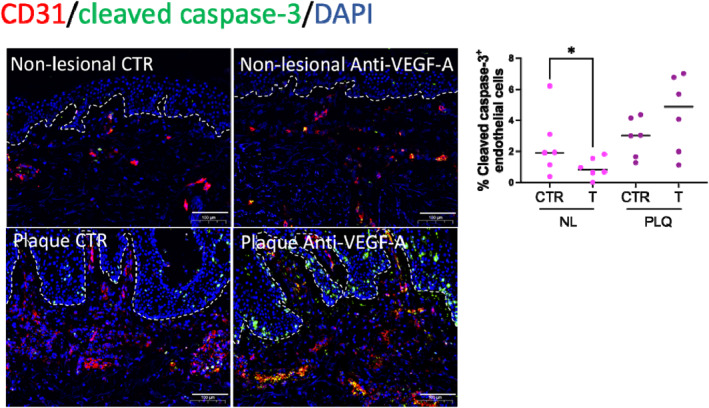
VEGF‐A inhibition downregulates EC apoptosis in psoriasis non‐lesional skin. EC apoptosis was assessed using double immunofluorescence staining CD31 for EC (red) and cleaved caspase‐3 for apoptosis (green). DAPI was used to stain the nuclei. VEGF‐A inhibition downregulated EC apoptosis in non‐lesional skin (0.81 [1.15]) compared to isotype control‐treated non‐lesional skin (1.92 [2.94]; *p < 0.05). Number of independent experiments *n* = 24 (1 punch biopsy per patient and treatment group). Data were presented as median and were analysed with Wilcoxon matched‐pairs signed rank test, two‐tailed. Scale bars = 100 μm. *p < 0.05. CTR, isotype control; EC, endothelial cell; NL, non‐lesional; PLQ, plaque; VEGF‐A, vascular endothelial growth factor A.

Lipids are known regulators of apoptotic cell death pathways,^41^, ^42^, ^43^, ^44^ so we interrogated whether VEGF‐A inhibition affected endothelial cell (EC) apoptosis in 72 h organ‐cultured non‐lesional skin ex vivo. The number of apoptotic ECs (CD31^+^ cleaved caspase‐3^+^) in bevacizumab‐treated non‐lesional skin (0.81 [1.15]) significantly decreased compared to isotype control‐treated non‐lesional skin (1.92 [2.94]; *p < 0.05; Figure 3). In contrast, the number of apoptotic ECs in bevacizumab‐treated plaque skin increased compared to isotype control‐treated plaque, although this did not reach significance (*p* = 0.15; Figure 3). VEGF‐A inhibition did not alter cleaved caspase‐3 expression in the stratum basale of non‐lesional or plaque skin (Figure S6).

### 3.8 VEGF‐A inhibition promoted ferroptosis in psoriasis plaque skin

We identified 13 activated pathways and 1 inhibited pathway in plaque in response to bevacizumab (*p*‐value <0.05; absolute *Z*‐score >2.0; Table S7). The enriched canonical pathways were assigned to the following categories: cellular stress and injury (ferroptosis, hypoxia‐inducible factor [HIF]1α, wound healing and antioxidant action of vitamin C signalling pathways), cellular immune response (phagosome formation, IFN, p38 MAPK and IL‐8 signalling pathways) and intracellular and second messenger signalling (eicosanoid signalling pathway). Others were assigned to fatty acid/lipid biosynthesis (superpathway of cholesterol biosynthesis and γ‐linoleate biosynthesis II) and fatty acid/lipid degradation (phospholipases). This further demonstrated the differential response to VEGF‐A inhibition in plaque and non‐lesional skin from patients with psoriasis.

## 4 DISCUSSION

In this study, aiming to unravel the molecular mechanisms of anti‐VEGF‐A treatment in psoriasis skin ex vivo, we investigated the early transcriptomic changes induced by bevacizumab in organ‐cultured psoriasis plaque and non‐lesional skin. VEGF‐A inhibition upregulated the expression of genes involved in lipid metabolism in psoriasis non‐lesional skin and, to a lesser extent, in plaque skin. VEGF‐A inhibition activated pathways associated with cellular stress and immune response in plaques of psoriasis.

Psoriasis‐associated altered circulating lipid levels^45^ and abnormal lipid composition in plaque and non‐lesional skin^3^ have been linked with decreased fatty acid elongation and disruption of the skin barrier function.^46^, ^47^, ^48^, ^49^ For instance, *ELOVL3*, *FADS1* and *FADS2*, which encode enzymes that participate in the elongation of long‐chain fatty acids are downregulated in plaque and non‐lesional skin compared to healthy skin.^34^, ^50^, ^51^, ^52^, ^53^, ^54^ The upregulation of genes such as *ELOVL3* and *PECR* in non‐lesional skin and *ELOVL3* and *FADS2* in plaque skin may improve lipid barrier function, suggesting that bevacizumab could potentially alleviate epidermal barrier dysfunction and limit disease progression in psoriasis. Alterations in lipid metabolism including increased lipid synthesis, fatty acid uptake and lipid droplet accumulation have been reported in cancer studies following anti‐angiogenic therapy.^55^, ^56^, ^57^ The oxygen deprivation induced by anti‐angiogenic therapy promotes a shift in lipid metabolism mediated by HIFs, which is essential for tumour survival in the reoxygenation phase^55^, ^58^ and this has been recognized as a mechanism for anti‐angiogenic drug resistance.^59^, ^60^ We have previously shown that VEGF‐A inhibition downregulates angiogenesis in plaque skin ex vivo.^30^ Therefore, transcriptional activation of HIF‐1α in response to VEGF‐A inhibition in plaques was expected, although this may pinpoint the activation of an early resistance mechanism to anti‐VEGF‐A treatment within plaques of psoriasis. However, while hypoxia may limit the beneficial effects of bevacizumab in plaque skin, VEGF‐A inhibition did not activate the HIF‐1α signalling pathway in non‐lesional skin. This observation is in keeping with a study that reports bevacizumab‐induced metabolic alterations in cell lines in normoxia.^61^ Taken together, our findings suggest that VEGF‐A inhibition may directly target cellular pathways involved in lipid metabolism in psoriasis non‐lesional skin, which potentially offers a treatment strategy to improve dyslipidaemia in psoriasis.

Ferroptosis activation in plaques of psoriasis may occur as a result of increased levels of oxidative stress along with impaired lipid catabolism due to hypoxia, making plaques of psoriasis particularly susceptible to reactive oxygen species (ROS) and lipid peroxidation formation.^62^, ^63^ Ferroptosis, an iron‐dependent type of programmed cell death characterized by ROS‐mediated lipid peroxidation,^64^, ^65^ has been linked to the therapeutic effect of classic chemotherapeutic drugs including cisplatin, paclitaxel, sulfasalazine and sorafenib.^66^, ^67^, ^68^, ^69^ However, ferroptosis is pro‐inflammatory,^70^, ^71^ contributes to EC activation^72^ and its role in the progression of psoriasis has become increasingly recognized.^73^, ^74^ For instance, ferroptosis inhibition suppressed psoriasis‐like inflammation in the imiquimod mouse model of psoriasis.^75^ Whether the activation of ferroptosis in response to VEGF‐A inhibition is beneficial in psoriasis remains to be determined. However, given the association between ferroptosis and elevated intracellular iron levels,^76^ it may be worth considering monitoring iron levels during anti‐angiogenic therapy.

We previously showed that VEGF‐A inhibition induced EC apoptosis in healthy skin in 72 h ex vivo organ culture,^33^ and here, we observed that VEGF‐A inhibition downregulated EC apoptosis in psoriasis non‐lesional skin. Various mechanisms of survival in response to anti‐angiogenic therapy have been described including autophagy, a type of programmed cell death that can be activated by cellular stress.^77^, ^78^ Bevacizumab induced autophagy in glioblastoma cells and colorectal cancer cells in vitro^79^, ^80^ and inhibition of bevacizumab‐induced autophagy led to an increase in apoptosis.^80^, ^81^ The identification of IRF1, a dual regulator of autophagy and apoptosis in response to bevacizumab treatment,^79^ as one of the top activated upstream regulators in non‐lesional skin, re‐enforces the hypothesis that IRF1 mediates the anti‐apoptotic effect of bevacizumab in non‐lesional psoriasis skin. Further studies are needed to validate this hypothesis.

This study had limitations. First, a limited number of biopsies and donors were available. However, we strengthened our RNA sequencing data analysis using IPA to compare our data with publicly available curated datasets. Second, the transcriptional analysis shows changes in the mRNA level of lipid synthesis. We suggest confirming the effect of VEGF‐A inhibition on lipid expression using lipidomic analysis in skin biopsies of patients treated with bevacizumab for diseases such as cancer or ophthalmological diseases. However, our histological data of VEGF‐A inhibition in non‐lesional skin supports our main findings and provides a model to validate our results further. Lastly, our investigation is limited by the ex vivo nature of skin organ culture. Nevertheless, this study provides proof‐of‐principle of a valuable tool for the transcriptional analysis of the effects of VEGF‐A inhibition in the skin.

In conclusion, the early effect of anti‐VEGF‐A treatment in modulating lipid metabolism in psoriasis deserves further investigation as it may provide an opportunity for cardiovascular risk management in psoriasis. Translating these results into daily clinical practice may involve monitoring the lipid and iron profiles of patients during anti‐VEGF‐A treatment. New developments in anti‐VEGF‐A therapies aiming to overcome drug resistance may rely on combination or sequential administrative strategies.

## CONFLICT OF INTEREST STATEMENT

The authors declare no conflicts of interest.

## AUTHOR CONTRIBUTIONS

**Andrea Luengas‐Martinez**: Data curation (lead); formal analysis (lead); investigation (lead); methodology (lead); writing—original draft (lead). **Dina Ismail**: Investigation (supporting); methodology (supporting); writing—review & editing (supporting). **Ralf Paus**: Conceptualization (supporting); data curation (supporting); formal analysis (supporting); funding acquisition (supporting); investigation (supporting); methodology (supporting); project administration (supporting); resources (supporting); supervision (supporting); writing—review & editing (supporting). **Helen S. Young**: Conceptualization (lead); data curation (supporting); formal analysis (supporting); funding acquisition (lead); investigation (supporting); methodology (supporting); project administration (lead); resources (lead); supervision (lead); writing—review & editing (lead).

## ETHICS STATEMENT

The study was approved by the UK Health Research Authority (15/NW/0585, 09/H/101143) and adhered to the Declaration of Helsinki Guidelines.

## PATIENT CONSENT

All patients gave written and informed consent.

## Supporting information

Figures S1–S6

Tables S1–S7

## Data Availability

No datasets were generated or analysed during the current study.
