# Supplementary material for: Vascular endothelial growth factor A inhibition remodels the transcriptional signature of lipid metabolism in psoriasis non‐lesional skin in 12 h ex vivo culture
Source: Skin Health Dis. 2024 Oct 26;4(6):e471. doi: 10.1002/ski2.471 (PMC11608907; doi:10.1002/ski2.471)
Supplement: Supplementary file 1 — Figures S1–S6 [file SKI2-4-e471-s001.docx]

Supplementary figures


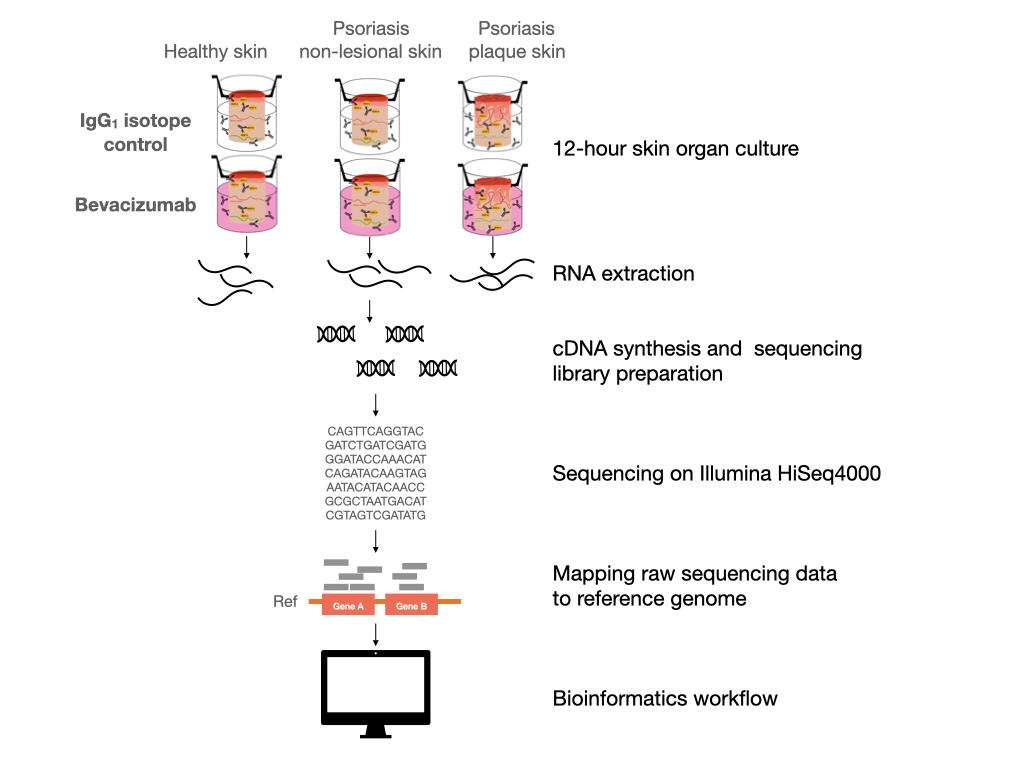


Figure S1 Workflow for RNA-Seq and data analysis

Skin biopsies from healthy controls and non-lesional and plaque skin from patients with psoriasis were incubated for 12 hours with 0.8 mg/ml of bevacizumab or with 0.8 mg/ml of human IgG_1_ isotype control. Following RNA extraction, complementary DNA (cDNA) libraries were prepared and RNA sequencing was performed in an Illumina HiSEq4000 instrument. Differential gene expression was performed using DESeq. Gene ontology (GO) analysis and Ingenuity Pathway Analysis (IPA) were used to identify enriched biological processes and enriched canonical pathways.

**Figure S2 Bevacizumab blocked free VEGF-A in organ culture**

VEGF-A was measured in the culture supernatant of organ-cultured healthy, non-lesional and plaque skin after 12 hours using an ELISA. VEGF-A was not detectable in the organ culture supernatant of healthy, non-lesional and plaque skin after 12 hours. The levels of VEGF-A in organ culture supernatant of isotype control-treated plaque control (589.9 [286.7]; p<0.01) were higher than in isotype control-treated non-lesional (0 [208.7]; *p*<0.01) and isotype control-treated healthy skin [107.7 (125.41); *p*<0.01]. * *p <* 0.05, ** p < 0.01. CTR: isotype control; H: healthy; NL: non-lesional; PLQ: plaque; T: treated with bevacizumab.

**Figure S3 Variation between samples was primarily due to disease status**

(**A**) Two major clusters emerged from principal component analysis (PCA). Principal component 1 (39 % of variance) separated samples based on disease status, where plaque control and treated skin samples clustered together and separated from non-lesional control and treated and healthy control and treated skin samples. Shapes represent disease status; size represents treatment status and the colours represent the different donors.


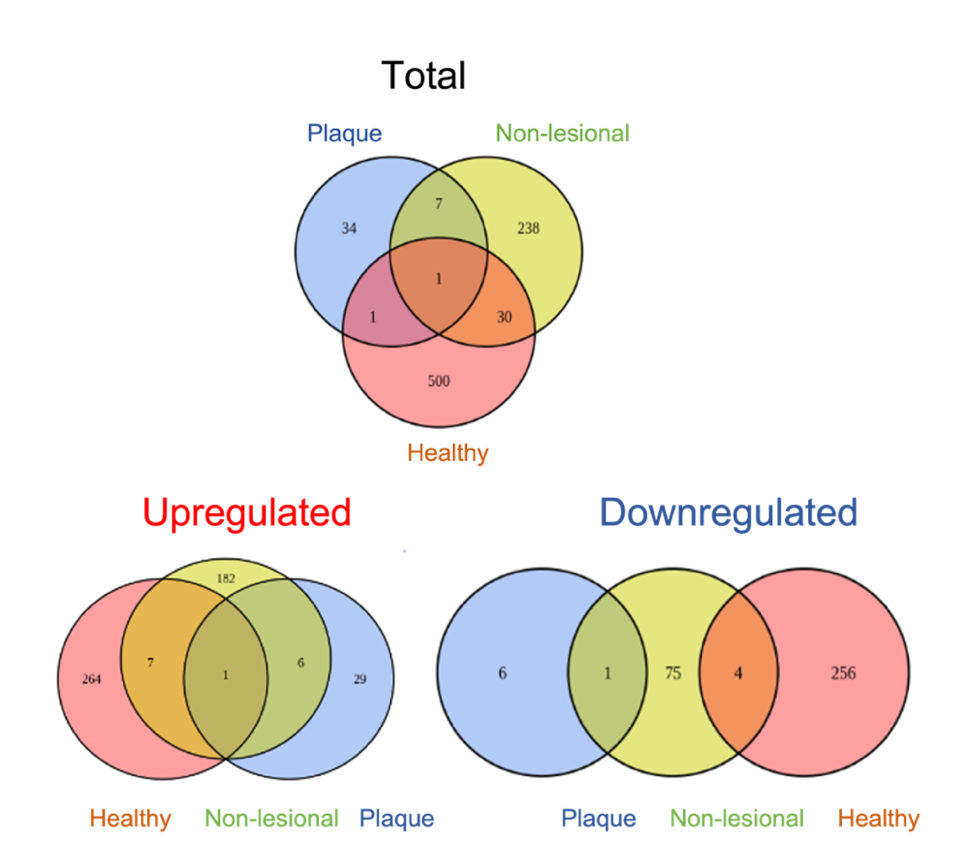


Figure S4 Bevacizumab-induced differential gene expression in healthy, psoriasis non-lesional and psoriasis plaque skin *ex vivo*

Venn diagram displaying the number of total and overlapping significant differentially expressed genes (DEGs) in bevacizumab-treated samples compared to control within each group: healthy, non-lesional and plaque skin from patients with psoriasis with an adjusted *p*-value<0.1. There was an overlap of 30 DEGs in non-lesional and healthy skin; and an overlap of 7 DEGs between plaque and non-lesional. One gene was upregulated in plaque skin and downregulated in healthy skin. One gene was differentially expressed in the three groups. 34 transcripts were solely differentially expressed in plaque skin, 238 in non-lesional skin and 500 in healthy skin in response to bevacizumab.


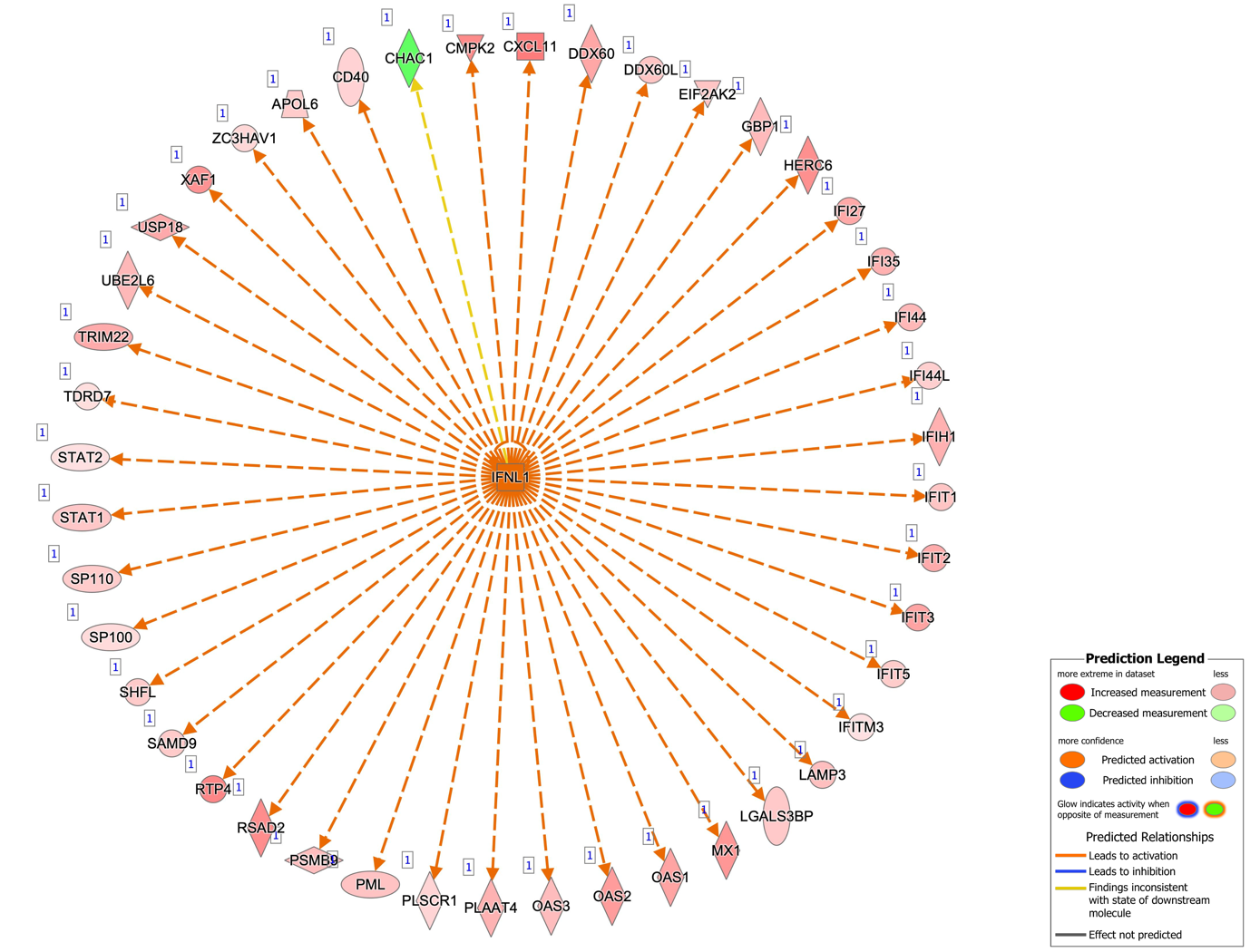


**Figure S5 IFNL1 target genes in the dataset**

Ingenuity Pathway Analysis identified 44 genes targeted by IFNL1 in non-lesional psoriasis skin.

**Figure S6 VEGF-A inhibition did not alter cleaved caspase-3 expression in the stratum basale**

Cleaved caspase-3 expression was assessed using immunofluorescence staining cleaved caspase-3 for apoptosis. Number of independent experiments n=24. Data were presented as median and were analysed with Wilcoxon matched-pairs signed rank test, two-tailed. CTR: isotype control; NL: non-lesional; PLQ: plaque.
