# Supplementary material for: Vascular endothelial growth factor A inhibition remodels the transcriptional signature of lipid metabolism in psoriasis non‐lesional skin in 12 h ex vivo culture
Source: Skin Health Dis. 2024 Oct 26;4(6):e471. doi: 10.1002/ski2.471 (PMC11608907; doi:10.1002/ski2.471)
Supplement: Supplementary file 2 — Tables S1–S7 [file SKI2-4-e471-s002.docx]

Supplementary Tables

Table S1 Overlap between differentially expressed genes by bevacizumab.

| Overlap between: | Gene | **Plaque** | | | **Non-lesional** | | |  | **Healthy** | |
| --- | --- | --- | --- | --- | --- | --- | --- | --- | --- | --- |
|  |  | Log2FC | Effect | Adjusted p-value | Log2FC | Effect | Adjusted p-value | Log2FC | Effect | Adjusted p-value |
| **Plaque and non-lesional** | *ELOVL3* | 1.42 | Up | 0.06 | 2.22 | Up | 0.07 |  |  | |
|  | *FBP1* | 1.29 | Up | 0.02 | 2.1 | Up | 0.000004 |  |  |  |
|  | *PECR* | 0.88 | Up | 0.04 | 1.33 | Up | 0.008 |  |  |  |
|  | *ATP12A* | 0.64 | Up | 0.02 | 1 | Up | 0.02 |  |  |  |
|  | *DEFB4A* | 0.58 | Up | 0.006 | 0.9 | Up | 0.008 |  |  |  |
|  | *GM2A* | 0.43 | Up | 0.007 | 0.72 | Up | 0.015 |  |  |  |
|  | *SPINK7* | -0.85 | Down | 0.04 | -1.05 | Down | 0.014 |  |  |  |
| **Plaque and healthy** | *C3* | 0.37 | Up | 0.02 |  |  | | -0.37 |  | 0.06 |
| **Plaque, non-lesional and healthy** | *RSAD2* | 0.6 | Up | 0.04 | 0.84 |  | 0.06543 | 0.69 |  | 0.0001 |
| **Non-lesional and healthy** | *ARSF* |  | | | 1.52 | Up | 0.02 | 1.01 | Up | 0.01 |
|  | *CAPN8* |  |  |  | 1.49 | Up | 0.03 | -1.3 | Down | 0.001 |
|  | *ABCA4* |  |  |  | 1.31 | Up | 0.05 | -1.72 | Down | 0.008 |
|  | *PRUNE2* |  |  |  | 1.14 | Up | 0.02 | -0.73 | Down | 0.06 |
|  | *RARRES1* |  |  |  | 1.13 | Up | 0.01 | -0.97 | Down | 0.005 |
|  | *PADI1* |  |  |  | 1.03 | Up | 0.01 | -1.1 | Down | 0.0009 |
|  | *CA6* |  |  |  | 0.96 | Up | 0.04 | -2.34 | Down | 0.06 |
|  | *HERC6* |  |  |  | 0.82 | Up | 3.96E-08 | 0.46 | Up | 0.009 |
|  | *MX1* |  |  |  | 0.76 | Up | 4.42E-09 | 0.44 | Up | 0.05 |
|  | *OAS2* |  |  |  | 0.73 | Up | 0.00001 | 0.45 | Up | 0.01 |
|  | *KRT15* |  |  |  | 0.72 | Up | 0.06 | -1.1 | Down | 0.06 |
|  | *DPYSL3* |  |  |  | 0.69 | Up | 0.006 | -0.4 | Down | 0.04 |
|  | *TPM1* |  |  |  | 0.67 | Up | 0.008 | -0.62 | Down | 0.009 |
|  | *MSRB3* |  |  |  | 0.61 | Up | 0.06 | -0.5 | Down | 0.006 |
|  | *PALMD* |  |  |  | 0.57 | Up | 0.04 | -0.62 | Down | 0.02 |
|  | *TNS1* |  |  |  | 0.49 | Up | 0.05 | -0.47 | Down | 0.0009 |
|  | *OAS3* |  |  |  | 0.49 | Up | 0.002 | 0.3 | Up | 0.07 |
|  | *LTBP2* |  |  |  | 0.47 | Up | 0.03 | -0.51 | Down | 0.001 |
|  | *LAMP3* |  |  |  | 0.47 | Up | 0.01 | 0.29 | Up | 0.05 |
|  | *DDAH1* |  |  |  | 0.47 | Up | 0.06 | -0.48 | Down | 0.07 |
|  | *BOC* |  |  |  | 0.36 | Up | 0.07 | -0.68 | Down | 0.0001 |
|  | *HSPA8* |  |  |  | 0.25 | Up | 0.02 | 0.33 | Up | 0.06 |
|  | *H2BC5* |  |  |  | -0.42 | Down | 0.08 | -0.41 | Down | 0.02 |
|  | *H1-0* |  |  |  | -0.53 | Down | 3.07E-10 | -0.3 | Down | 0.01 |
|  | *LCE1C* |  |  |  | -0.54 | Down | 0.05 | 0.29 | Up | 0.04 |
|  | *LCE6A* |  |  |  | -0.55 | Down | 0.05 | 0.24 | Up | 0.09 |
|  | *H1-10* |  |  |  | -0.56 | Down | 0.006 | -0.49 | Down | 0.00004 |
|  | *KRT78* |  |  |  | -0.57 | Down | 0.06 | 0.38 | Up | 0.01 |
|  | *LCEP3* |  |  |  | -0.61 | Down | 0.02 | 0.32 | Up | 0.03 |
|  | *BAMBI* |  |  |  | -0.93 | Down | 0.07 | -0.79 | Down | 0.01 |

**Table S2 Differentially expressed genes in plaque skin from patients with psoriasis.**

| Upregulated genes | | | Downregulated genes | | |
| --- | --- | --- | --- | --- | --- |
| Gene | Log2FC | Adjusted p-value | Gene | Log2FC | Adjusted p-value |
| *SLCO4C1* | 1.57 | 0.02 | *LINC02404* | -1.9 | 0.01 |
| *ELOVL3* | 1.42 | 0.06 |  |  |  |
| *COCH* | 1.31 | 0.01 |  |  |  |
| *FADS2* | 1.29 | 0.02 |  |  |  |
| *FBP1* | 1.29 | 0.02 |  |  |  |

Differentially expressed genes (DEGs) with p-value < 0.1 and log2FC > 1 were considered significant. The DEGs were sorted based on the Log2FC.

**Table S3 Top 30 differentially expressed genes in non-lesional psoriasis skin.**

| Upregulated genes | | | Downregulated genes | | |
| --- | --- | --- | --- | --- | --- |
| Gene | Log2FC | Adjusted p-value | Gene | Log2FC | Adjusted p-value |
| *PADI4* | 3.17 | 0.02 | *LINC02527* | -1.91 | 0.0006 |
| *SEC14L4* | 3.08 | 0.00001 | *SPINK7* | -1.05 | 0.01 |
| *THRSP* | 2.95 | 2.08E-07 | *ENSG00000275216* | -1 | 0.02 |
| *MOGAT2* | 2.84 | 3.96E-08 |  |  |  |
| *IL12A-AS1* | 2.79 | 0.04 |  |  |  |
| *MOGAT1* | 2.78 | 0.002 |  |  |  |
| *ACSM6* | 2.63 | 0.00009 |  |  |  |
| *CUX2* | 2.58 | 2.01E-07 |  |  |  |
| *PCK1* | 2.58 | 0.05 |  |  |  |
| *METTL7B* | 2.47 | 0.05 |  |  |  |
| *LINC01612* | 2.43 | 0.01 |  |  |  |
| *SLC25A18* | 2.34 | 0.008 |  |  |  |
| *AGR2* | 2.3 | 0.0003 |  |  |  |
| *RNASE13* | 2.23 | 0.06 |  |  |  |
| *ELOVL3* | 2.22 | 0.07 |  |  |  |
| *CYP4F8* | 2.17 | 0.01 |  |  |  |
| *FBP1* | 2.1 | 4.78E-06 |  |  |  |
| *LINC02240* | 2.06 | 0.01 |  |  |  |
| *DHRS2* | 2.03 | 0.001 |  |  |  |
| *LINC00885* | 2.01 | 0.07 |  |  |  |
| *CAMP* | 2.01 | 0.008 |  |  |  |
| *LINC02568* | 1.97 | 0.07 |  |  |  |
| *TMEM63C* | 1.92 | 0.016 |  |  |  |
| *HGD* | 1.92 | 0.04 |  |  |  |
| *APOC1* | 1.88 | 0.001 |  |  |  |
| *CPB2-AS1* | 1.88 | 0.09 |  |  |  |
| *HP* | 1.83 | 0.07 |  |  |  |
| *ROS1* | 1.77 | 0.06 |  |  |  |
| *ADGRL3* | 1.75 | 0.01 |  |  |  |
| *KRT4* | 1.75 | 0.03 |  |  |  |

Differentially expressed genes (DEGs) with p-value < 0.1 and log2FC > 1 were considered significant. The DEGs were sorted based on the Log2FC.

**Table S4 Top 30 differentially expressed genes in healthy skin**

| Upregulated genes | | | Downregulated genes | | |
| --- | --- | --- | --- | --- | --- |
| Gene | Log2FC | Adjusted p-value | Gene | Log2FC | Adjusted p-value |
| *ZFP57* | 1.47 | 0.022264 | *KRT85* | -10.14 | 0.044481 |
| *ENSG00000224721* | 1.02 | 0.066637 | *KRT81* | -4.92 | 0.086916 |
| *ARSF* | 1.01 | 0.012047 | *KRTAP5-10* | -4.85 | 0.00002 |
|  |  |  | *LYG2* | -3.8 | 0.08 |
|  |  |  | *B4GALNT2* | -2.99 | 6.19E-06 |
|  |  |  | *NPPC* | -2.45 | 0.002 |
|  |  |  | *CA6* | -2.34 | 0.06 |
|  |  |  | *EMILIN3* | -2.32 | 0.01 |
|  |  |  | *ABCA4* | -1.72 | 0.008 |
|  |  |  | *ALDH1L1* | -1.71 | 0.01 |
|  |  |  | *TRIL* | -1.64 | 0.08 |
|  |  |  | *SMOC1* | -1.61 | 0.02 |
|  |  |  | *SHH* | -1.54 | 0.09 |
|  |  |  | *COPG2IT1* | -1.51 | 0.06 |
|  |  |  | *HMGCS2* | -1.51 | 0.005 |
|  |  |  | *WNK4* | -1.5 | 0.06 |
|  |  |  | *SHISA2* | -1.4 | 0.002 |
|  |  |  | *NTRK3* | -1.37 | 0.02 |
|  |  |  | *CAPN8* | -1.3 | 0.001 |
|  |  |  | *CRACR2B* | -1.27 | 0.00008 |
|  |  |  | *CECR2* | -1.23 | 0.05 |
|  |  |  | *SLC40A1* | -1.22 | 5.36E-06 |
|  |  |  | *COL9A2* | -1.21 | 0.02 |
|  |  |  | *MUCL1* | -1.2 | 0.09 |
|  |  |  | *ALPL* | -1.19 | 0.09 |
|  |  |  | *CMYA5* | -1.14 | 0.03 |
|  |  |  | *ANOS1* | -1.13 | 0.06 |
|  |  |  | *CDK18* | -1.13 | 0.006 |
|  |  |  | *SHANK2* | -1.12 | 0.01 |
|  |  |  | *KRT15* | -1.1 | 0.06 |

Differentially expressed genes (DEGs) with p-value < 0.1 and log2FC > 1 were considered significant. The DEGs were sorted based on the Log2FC.

Table S5 Canonical pathways affected in bevacizumab-treated vs control-treated non-lesional psoriasis skin.

| **Ingenuity Canonical Pathways** | **-log(p-value)** | **z-score** | **Genes** |
| --- | --- | --- | --- |
| Fatty Acid β-oxidation I | 5.98 | 3.162 | *ACAA2, ACADM, ACSBG1, ACSF2, ACSL1, ACSL3, ACSL5, ECHDC3, HADH, HSD17B4, IVD, SCP2* |
| Interferon Signalling | 5.98 | 2.887 | *BAX, IFI35, IFIT1, IFIT3, IFITM3, JAK2, MX1, OAS1, PSMB8, STAT1, STAT2, TAP1* |
| Superpathway of Cholesterol Biosynthesis | 5.23 | 3 | *ACAA2, ACAT2, DHCR24, DHCR7, FDPS, HMGCS2, MSMO1, MVD, SC5D, TM7SF2* |
| γ-linolenate Biosynthesis II (Animals) | 5.03 | 2.646 | *ACSBG1, ACSF2, ACSL1, ACSL3, ACSL5, CYB5A, FADS1, FADS2* |
| Ethanol Degradation II | 4.54 | 3.162 | *ACSL1, ACSS3, ADH5, AKR1A1, ALDH1A1, ALDH1A2, ALDH3A2, ALDH7A1, ALDH9A1, DHRS2* |
| Ethanol Degradation IV | 4.16 | 2.828 | *ACSL1, ACSS3, ALDH1A1, ALDH1A2, ALDH3A2, ALDH7A1, ALDH9A1, CAT* |
| Role of Hypercytokinemia/ hyperchemokinemia in the Pathogenesis of Influenza | 4.12 | 2.5 | *AREG, CASP1, EIF2AK2, IFIT2, IFIT3, IL1A, IL37, MX1, OAS1, OAS2, OAS3, PYCARD, RSAD2, STAT1, STAT2, TLR3* |
| Leucine Degradation I | 4.01 | 2.236 | *ACADM, BCAT2, HMGCL, IVD, MCCC1* |
| Apelin Adipocyte signalling Pathway | 3.82 | 2.496 | *ADCY5, ADCY7, CAT, GPX2, GPX3, GPX4, GSTK1, LIPE, MAPK11, MGST1, MGST2, MGST3, PPARGC1A, PTGES, RAC3, UCP1* |
| Valine Degradation I | 3.71 | 2.236 | *ACAD8, BCAT2, BCKDHB, ECHDC3, HIBADH, HIBCH, HSD17B4* |
| Glutathione Redox Reactions I | 3.64 | 2.646 | *GPX2, GPX3, GPX4, GSTK1, MGST1, MGST2, MGST3, PTGES* |
| Noradrenaline and Adrenaline Degradation | 3.44 | 3 | *ADH5, AKR1A1, ALDH1A1, ALDH1A2, ALDH3A2, ALDH7A1, ALDH9A1, DHRS2, IL4I1* |
| Cholesterol Biosynthesis I | 3.10 | 2.236 | *DHCR24, DHCR7, MSMO1, SC5D, TM7SF2* |
| Cholesterol Biosynthesis II (via 24,25-dihydrolanosterol) | 3.10 | 2.236 | *DHCR24, DHCR7, MSMO1, SC5D, TM7SF2* |
| Cholesterol Biosynthesis III (via Desmosterol) | 3.10 | 2.236 | *DHCR24, DHCR7, MSMO1, SC5D, TM7SF2* |
| Xenobiotic Metabolism AHR Signaling Pathway | 3.02 | 2.673 | *CYB5A, FADS1, FADS2, SCD* |
| Tryptophan Degradation X (Mammalian, via Tryptamine) | 2.97 | 2.646 | *CASP1, CASP5, CD40, CIITA, DEFB4A/DEFB4B, FCGR2B, JAK2, NLRC5, NLRP10, TLR3, TREM1* |
| Stearate Biosynthesis I (Animals) | 2.78 | 2.828 | *ACOT1, ACOT11, ACOT2, ACOT7, ACSBG1, ACSF2, ACSL1, ACSL3, ACSL5, ASPG, DHCR24* |
| Fatty Acid Activation | 2.77 | 2 | *ACSBG1, ACSF2, ACSL1, ACSL3, ACSL5* |
| Superpathway of Geranylgeranyldiphosphate Biosynthesis I (via Mevalonate) | 2.39 | 2 | *ACAA2, ACAT2, FDPS, HMGCS2, MVD* |
| Mitochondrial L-carnitine Shuttle Pathway | 2.28 | 2 | *ACSBG1, ACSF2, ACSL1, ACSL3, ACSL5* |
| Oleate Biosynthesis II (Animals) | 2.16 | 2 | *CYB5A, FADS1, FADS2, SCD* |
| TREM1 signalling | 2.09 | 2.714 | *CASP1, CASP5, CD40, CIITA, DEFB4A/DEFB4B, FCGR2B, JAK2, NLRC5, NLRP10, TLR3, TREM1* |
| Histamine Degradation | 2.08 | 2.236 | *ALDH1A1, ALDH1A2, ALDH3A2, ALDH7A1, ALDH9A1* |
| Fatty Acid α-oxidation | 2.08 | 2.236 | *ALDH1A1, ALDH1A2, ALDH3A2, ALDH7A1, ALDH9A1* |
| Retinoic acid Mediated Apoptosis signalling | 1.93 | 2.828 | *CRABP2, PARP10, PARP12, PARP14, PARP3, PARP4, PARP8, PARP9, TNFSF10* |
| Dopamine Degradation | 1.88 | 2.449 | *ALDH1A1, ALDH1A2, ALDH3A2, ALDH7A1, ALDH9A1, IL4I1* |
| Death Receptor signalling | 1.78 | 2.309 | *ACTC1, CASP10, HSPB7, PARP10, PARP12, PARP14, PARP3, PARP4, PARP8, PARP9, SPTAN1, TNFSF10* |
| Production of Nitric Oxide and Reactive Oxygen Species in Macrophages | 1.78 | 2.183 | *APOC1, APOL1, ARG2, CAT, DIRAS3, FOS, JAK2, MAP3K9, MAPK11, PCYOX1, PIK3C2G, PRKCB, PTPA, RAC3, RHOBTB1, RHOD, RHOT1, S100A8, SERPINA1, STAT1* |
| Gluconeogenesis I | 1.68 | 2.236 | *ENO2, FBP1, MDH1, ME1, PGK1* |
| Oxidative Phosphorylation | 1.67 | 3.606 | *ATP5F1A, ATP5MC3, ATP5PD, COX5B, CYB5A, NDUFA4, NDUFA5, NDUFB3, NDUFB5, NDUFS5, SDHC, UQCRB, VPS9D1* |
| Cardiac Hypertrophy Signalling (Enhanced) | 1.65 | 2.214 | *ADCY5, ADCY7, ADRB2, BMPR2, CALML5, CAMK2B, CNTF, DIAPH1, DVL1, EIF2B4, EIF4EBP1, GSK3A, HDAC4, HDAC7, HSPB7, IL15, IL15RA, IL17RE, IL1A, IL1RL2, IL20RA, IL22RA2, IL2RB, IL31RA, IL37, ITGA7, ITGA8, JAK2, MAP3K20, MAP3K9, MAPK11, MKNK2, MYOCD, PDE1B, PDE6A, PIK3C2G, PLCE1, PLN, PRKCB, PRKG1, RALB, RRAS2, RYR2, SMPDL3B, TNFSF10, WNT11* |
| Phospholipases | 1.64 | 3 | *GPLD1, PLA1A, PLA2G7, PLA2R1, PLAAT3, PLAAT4, PLCE1, PLD3, PNPLA3* |
| Glycolysis I | 1.62 | 2.236 | *ENO2,FBP1,PFKL,PGK1,PKLR* |
| Eicosanoid Signaling | 1.60 | 2.236 | *ALOX12, ALOX15B, PLA2G7, PLA2R1, PLAAT3, PLAAT4, PNPLA3, PTGER3, PTGES* |
| Endothelin-1 Signaling | 1.49 | 3.3 | *ADCY5, ADCY7, CASP1, CASP10, CASP5, FOS, GPLD1, MAPK11, PIK3C2G, PLA2G7, PLA2R1, PLAAT3, PLAAT4, PLCE1, PLD3, PNPLA3, PRKCB, RALB, RRAS2* |
| Serotonin Degradation | 1.49 | 3 | *ADH5, AKR1A1, ALDH1A1, ALDH1A2, ALDH3A2, ALDH7A1, ALDH9A1, DHRS2, IL4I1* |
| Fcγ Receptor-mediated Phagocytosis in Macrophages and Monocytes | 1.49 | 2.111 | *ACTC1, FGR, FYB1, FYN, GAB2, GPLD1, PIK3C2G, PLD3, PRKCB, RAC3, VAV3* |
| Integrin Signaling | 1.34 | 2.324 | *ACTC1, CAPN8, DIRAS3, FYN, ITGA7, ITGA8, MPRIP, MYL9, MYLK, PFN2, PIK3C2G, PPP1R12B, RAC3, RALB, RHOBTB1, RHOD, RHOT1, RRAS2, TSPAN2, TSPAN6* |
| Antioxidant Action of Vitamin C | 1.32 | -3 | *GPLD1, JAK2, MAPK11, NXN, PLA2G7, PLA2R1, PLAAT3, PLAAT4, PLCE1, PLD3, PNPLA3, SLC2A14* |

–log(*p*-value) >1.3 reflects a significant association between the canonical pathway and its involved genes; positive and negative Z-scores are considered activated and inhibited, respectively. Pathways with a Z-score more than 2.0 were considered for significant activation or less than -2.0 for significant inhibition status in the pathway analysis. The pathways were sorted based on the –log (*p*-value).

Table S6 Top 10 upstream regulators in non-lesional skin samples treated with bevacizumab.

| Upstream regulator | Expr log ratio | Molecule type | Activation Z-score^1^ | p-value of overlap^2^ |
| --- | --- | --- | --- | --- |
| IFNL1 | -1.498 | cytokine | 6.26 | 4.95E-29 |
| Interferon alpha |  | group | 5.99 | 4.92E-25 |
| NKX2-3 | 0 | transcription regulator | -4.3 | 1.02E-24 |
| IRF7 | -0.045 | transcription regulator | 6.9 | 1.68E-22 |
| IFNG | 0.2 | cytokine | 7.8 | 4.98E-22 |
| NONO | -0.043 | transcription regulator | 6.46 | 8.03E-22 |
| IRF1 | 0.238 | transcription regulator | 5.8 | 8.81E-22 |
| IRGM | 1.437 | enzyme | -5.34 | 1.67E-21 |
| STAT1 | 0.411 | transcription regulator | 7 | 3.27E-21 |
| IFNA2 | 0.602 | cytokine | 6.85 | 3.9E-21 |
| lipopolysaccharide |  | chemical drug | 6.49 | 5.43E-20 |

1. Positive and negative z-scores are considered activated and inhibited, respectively. 2. A p-value of overlap indicates whether there is a statistically significant interaction between an upstream regulator and its regulated genes. A p-value of overlap <0 .01 is considered statistically significant. The regulators were sorted based on the p-value of overlap.

Table S7 Canonical pathways enriched in bevacizumab-treated vs control-treated plaque skin.

| **Ingenuity Canonical Pathways** | **-log(*p*-value)** | **z-score** | **Genes** |
| --- | --- | --- | --- |
| Ferroptosis Signalling Pathway | 5.12 | 2.673 | *ALOX12B, ALOX15B, ANGPTL4, CTSB, DPP4, FTH1, FTL, H2AX, H2AZ1, HMOX1, SAT1, SLC11A2, SLC38A1, SP1, TF* |
| HIF1α signalling | 4.37 | 3.153 | *CAMK2D, FLT1 ,FOXP3, HK2, HMOX1, MMP10, MMP12, MMP14, MMP19, MMP2, MMP9, NOS2, RAC2, RPS6KB1, SAT1, SLC2A3, SLC2A5, TF* |
| Phagosome Formation | 3.80 | 2.271 | *ADCYAP1R1, ADRB2, APBB1IP, APLNR, BDKRB2, C3, CCR1, CD209, CD36, EDNRB, FCGR2A, FCGR2B, GPRC5A, HCK, HMOX1, IGHG4, ITGA10, ITGA11, ITGA6, ITGAX, ITGB7, ITPR2, LCK, MAPK7, MRC1, NPY1R, P2RY1, PLA2G2F, PLA2G4D, PLA2G7, PLD3, PNPLA3, PTAFR, PTGFR, RAC2, RPS6KB1, SCARA5, VAV3* |
| Interferon Signalling | 3.25 | 2.449 | *IFI35, IFIT3, IFNGR1, MX1, PIAS1, STAT1* |
| Eicosanoid Signalling | 3.10 | 2 | *ALOX12B, ALOX15B, PLA2G2F, PLA2G4D, PLA2G7, PNPLA3, PTGFR, PTGIS* |
| Superpathway of Cholesterol Biosynthesis | 2.86 | 2.236 | *ACAT2, DHCR24, DHCR7, MVD, MVK* |
| γ-linolenate Biosynthesis II (Animals) | 2.71 | 2 | *ACSL1, ACSL5, FADS1, FADS2* |
| Wound Healing Signalling Pathway | 2.50 | 2.5 | *ACTA2, CMA1, COL11A2, COL13A1, COL5A2, COL6A3, IFNGR1, IL36A, IL36RN, ITGA6, MMP10, MMP9, STAT1, TGFB3, TNFRSF1B, TRPV1* |
| Antioxidant Action of Vitamin C | 2.25 | -2.449 | *GSTO1, HMOX1, PLA2G2F, PLA2G4D, PLA2G7, PLD3, PNPLA3, SLC2A3, SLC2A5* |
| p38 MAPK Signalling | 2.06 | 2.828 | *DDIT3, IL36A, IL36RN, PLA2G2F, PLA2G4D, RPS6KB1, STAT1, TGFB3, TNFRSF1B* |
| Role of Hypercytokinemia/  hyperchemokinemia in the Pathogenesis of Influenza | 1.89 | 2.646 | *EIF2AK2, IFIT3, IL36A, IL36RN, MX1, RSAD2, STAT1* |
| Phospholipases | 1.87 | 2.449 | *HMOX1, PLA2G2F, PLA2G4D, PLA2G7, PLD3, PNPLA3* |
| Tumour Microenvironment Pathway | 1.42 | 2.53 | *MMP10, MMP12, MMP14, MMP19, MMP2, MMP9, NOS2, SLC2A3, TGFB3, TIAM1* |
| IL-8 signalling | 1.35 | 2.111 | *ANGPT2, CXCR2, FLT1, HMOX1, ITGAX, MMP2,MMP9, PLD3, RAC2, RHOU, RPS6KB1* |

–log(*p*-value) >1.3 reflects a significant association between the canonical pathway and its involved genes; positive and negative Z-scores are considered activated and inhibited, respectively. Pathways with a Z-score more than 2.0 were considered for significant activation or less than -2.0 for significant inhibition status in the pathway analysis. The pathways were sorted based on the –log (*p*-value).
